# The Catabolite Repression Control Protein Crc Regulates the Type III Secretion System Through the Adenylate Cyclase CyaB in *Pseudomonas aeruginosa*

**DOI:** 10.3390/microorganisms13112587

**Published:** 2025-11-13

**Authors:** Liwen Yin, Xuetao Gong, Yiming Li, Junze Qu, Yu Zhang, Yongxin Jin, Shouguang Jin, Weihui Wu

**Affiliations:** State Key Laboratory of Medicinal Chemical Biology, Key Laboratory of Molecular Microbiology and Technology of the Ministry of Education, Department of Microbiology, College of Life Sciences, Nankai University, Tianjin 300071, China; yinliwen2022@163.com (L.Y.); 1120220627@mail.nankai.edu.cn (X.G.); mitchell.li@foxmail.com (Y.L.); junze0304@126.com (J.Q.); zyuu2025@163.com (Y.Z.); yxjin@nankai.edu.cn (Y.J.); nksjin@nankai.edu.cn (S.J.)

**Keywords:** *P. aeruginosa*, type III secretion system, Crc, CyaB

## Abstract

*Pseudomonas aeruginosa* is a versatile Gram-negative pathogen that causes various infections in humans. The bacterium possesses a type III secretion system (T3SS) to deliver cytotoxic effector proteins into host cells, which plays an important role in bacterial pathogenesis. The T3SS is regulated by the master regulator ExsA, whose expression is controlled by multiple pathways. Here, we demonstrate that the catabolite repression control protein Crc controls T3SS activity by modulating *exsA* expression. We find that mutation of *crc* reduces the intracellular cAMP level by 1.76-fold under T3SS-inducing conditions, leading to approximately 2-fold reduction of the *exsA* expression. Further investigation reveals that Crc affects the mRNA stability of *cyaB*, which encodes an adenylate cyclase involved in cAMP synthesis. The *cyaB* 5′-UTR is identified as a key region through which Crc affects its mRNA stability. Our study elucidates a novel regulatory mechanism by which Crc controls the T3SS through modulating *cyaB* mRNA stability and subsequent cAMP synthesis under T3SS-inducing conditions.

## 1. Introduction

*Pseudomonas aeruginosa* is an opportunistic Gram-negative pathogen that causes a variety of nosocomial infections in immunocompromised individuals [1,2]. The bacterium utilizes multiple virulence factors to establish chronic and acute infections [2,3]. The type III secretion system (T3SS) plays a critical role during acute infections [4,5]. It is a syringe-like apparatus that directly injects cytotoxic effector proteins into host cells to counteract immune responses and cause tissue damage [6,7,8].

The activation of T3SS is energetically costly, thus it is subject to tight regulation in response to environmental signals, such as low calcium and host cell contact [9]. ExsA is the master regulator that regulates all the T3SS genes. The *exsA* gene is localized in the *exsCEBA* operon [10,11]. Its transcription is driven by its own adjacent promoter and the *exsC* promoter, which are directly regulated by Vfr in combination with cAMP and ExsA, respectively [12,13]. In *P. aeruginosa*, intracellular cAMP is synthesized by adenylate cyclases CyaA/CyaB and degraded by the phosphodiesterase CpdA [14,15,16]. It has been demonstrated that CyaB plays a major role in the biosynthesis of cAMP [14]. Additionally, the *exsA* promoter is directly regulated by the transcription factors PsrA, Fis, and MvaT [17,18,19].

In *P. aeruginosa*, the Crc protein plays a crucial role in carbon catabolite repression (CCR), ensuring the hierarchical utilization of carbon sources [20]. In the presence of a preferred carbon source, Crc inhibits the use of less-preferred carbon sources by repressing the expression of corresponding enzymes at the posttranscriptional level [21,22]. Crc and the RNA chaperone Hfq form a complex that binds to target mRNAs and represses their translation [23]. The small RNA CrcZ binds to Hfq, alleviating CCR [24]. The expression of CrcZ is controlled by the two-component system CbrA/B [25]. Besides CCR, Crc also influences multiple pathways related to bacterial pathogenesis, such as motility [26], quorum sensing [27,28], biofilm formation [29,30], and the T3SS [22].

Yeung et al. demonstrated that a mutation of *crc* reduced bacterial cytotoxicity [31]. Transcriptomic and proteomic analyses revealed that Crc is required for the expression of the T3SS genes in *P. aeruginosa* [22,32]. Gil-Gil et al. demonstrated that a mutation of *crc* reduced the expression of *exsA* and the bacterial proton motive force (PMF) [33]. However, the mechanism of Crc-mediated regulation of the T3SS remains elusive. The objective of the present research was to determine the mechanism of Crc-mediated regulation on the T3SS and to provide insight on the interconnection between bacterial virulence and metabolism. In this work, we constructed a *crc* deletion mutant in the wild type reference *P. aeruginosa* strain PAK [34,35]. Our results revealed that Crc modulates the mRNA stability of *cyaB*, which then regulates the T3SS through the cAMP-Vfr pathway under T3SS-inducing conditions.

## 2. Materials and Methods

### 2.1. Bacterial Strains and Plasmids

The bacterial strains, plasmids, and primers utilized in this study are listed in Tables S1 and S2. *P. aeruginosa* and *E. coli* strains were grown in the Lysogeny broth (LB) medium with shaking at 200 rpm at 37 °C. Antibiotics were used at the following concentrations: for *P. aeruginosa*, tetracycline 50 μg/mL, carbenicillin 150 μg/mL, and gentamicin 50 μg/mL; for *E. coli*, tetracycline 10 μg/mL, kanamycin 25 µg/mL, gentamicin 10 μg/mL, and ampicillin 100 μg/mL.

### 2.2. Western Blot Assay

Overnight bacteria cultures were diluted into 3 mL fresh LB medium with or without 5 mM EGTA, and cultured to OD_600_ of 1.0. Bacterial cells and supernatants were collected following centrifugation at 12,000× *g* for 3 min. The samples were run in a 12% SDS-PAGE gel and then transferred onto a polyvinylidene difluoride (PVDF) membrane. The protein levels were determined using antibodies against ExoS, FLAG (Sigma, Livonia, MI, USA), His (Millipore, Burlington, MA, USA) or RpoA (Abcam, Cambridge, MA, USA), and HRP-conjugated anti-Rabbit IgG (Promega, Madison, WI, USA) or anti-Mouse IgG (Promega, USA). The signals were detected with an Immobilon Western Chemiluminescent HRP Substrate (Millipore, USA) and a Bio-Rad molecular imager (ChemiDocXRS+, Bio-Rad, Hercules, CA, USA).

### 2.3. Cytotoxicity Assay

The cytotoxicity assay was carried out as previously described with minor modification [36]. A549 cells were cultured in Roswell Park Memorial Institute (RPMI) 1640 medium containing 10% fetal bovine serum (FBS) in a 24-well plate at 37 °C in 5% CO_2_. Log growth phase bacteria (OD_600_ = 1.0) were resuspended in PBS and used to infect A549 cells at a multiplicity of infection (MOI) of 50. Two hours post infection, the dead cells were removed by washing with PBS and the remaining cells were stained with 0.25% crystal violet. After washed twice with PBS, the crystal violet that reflected the number of live cells was dissolved by a destaining buffer (40% methanol, 10% acetic acid) and measured at a wavelength of 595 nm using a Varioskan Flash microplate reader (Thermo Scientific, Waltham, MA, USA).

### 2.4. cAMP Assay

Intracellular cAMP concentration was measured as previously described [37]. Bacteria were grown in LB to an OD_600_ of 1.0. Then, 1.5 mL of the bacteria were centrifuged at 13,000× *g* for 2 min at 4 °C, then washed twice with cold 0.9 M NaCl. The pellets were resuspended in 100 μL of 0.1 M HCl, and incubated on ice for 10 min with a vortex. After centrifugation at 13,000× *g* for 5 min at 4 °C, the supernatant was used to measure cAMP concentration with an ELISA kit (Cayman, Ann Arbor, MI, USA) following the manufacturer’s protocol. The bacterial protein concentration was measured by a BCA assay (Beyotime Biotechnology, Shanghai, China). The cAMP levels were normalized by protein concentrations.

### 2.5. RNA Purification and Quantitative Real-Time PCR

RNA purification and quantitative real-time PCR were carried out following the manufacturer’s instructions with minor modifications as described below. Bacteria were grown in LB with or without 5 mM EGTA to OD_600_ of 1.0. Total RNA was isolated with a Bacteria Total RNA Kit (Zomanbio, Beijing, China). The DNA removal and cDNA synthesis were carried out using HiScript III RT SuperMix (+gDNA wiper) (Vazym, Nanjing, China). RT-qPCR was performed using PerfectStart^®^ Green qPCR SuperMix (Transgen, Beijing, China). The primers used in this study are listed in Table S2. The housekeeping ribosomal gene *rpsL* was used as the reference gene for normalization. The data are presented as the relative transcript abundance of the indicated genes compared to the internal control transcript for *rpsL* using the comparative Ct method (2^−∆∆CT^).

To test the mRNA stability, the bacteria were grown in LB with 5 mM EGTA to the OD_600_ of 1.0, followed by treatment with 200 μg/mL rifampicin. At each indicated time point after rifampicin treatment, qual numbers of bacterial cells were harvested and mixed with equal numbers of *gfp*-expressing *E. coli* cells. Then, the corresponding RNA was extracted for RT-qPCR with *gfp* as an internal control.

### 2.6. 5′ Rapid Amplification of cDNA Ends (5′ RACE) Assay

The 5′ RACE assay was carried out as previously described [38]. Total RNA extraction and cDNA synthesis were carried out as previously mentioned. PolyG was added to the 3′ end of the cDNA using a Terminal Deoxynucleotidyl Transferase Kit (Takara, Beijing, China). The product was used as a template to amplify the *cyaB* sequence using primers 5-AP and RACE-*cyaB*-R (Table S2). The product was purified and used as a template for a second round of amplification using the primers 5-NP and RACE-*cyaB*-R2 (Table S2). The product was then purified and sequenced to determine the transcription start site.

### 2.7. β-Galactosidase Assay

The β-galactosidase assay was carried out as described previously with minor modifications [39]. Bacteria from 1 mL of culture were collected by centrifugation and resuspended in 1.5 mL Z-buffer (60 mM Na_2_HPO_4_, 40 mM NaH_2_PO_4_, 50 mM β-mercaptoethanol, 10 mM KCl, 1 mM MgSO_4_). Then, 1 mL of the resuspension was used to measure OD_600_. To the remaining 500 μL resuspension, 10 μL chloroform and 10 μL 0.1% SDS were added. After vortexing for 10 s, 100 μL ONPG (4 mg/mL) was added. The mixture was incubated at 37 °C, and 500 μL 1 M Na_2_CO_3_ was added to stop the reaction. The reaction time was recorded. After centrifugation at 16,000× *g* for 5 min, OD_420_ was measured. The β-galactosidase activity (Miller units) was calculated as 1000 × OD_420_/T_min_/V_mL_/OD_600_.

### 2.8. Data Analysis

Statistical analyses were performed using GraphPad Prism 8 (GraphPad Software, Boston, MA, USA). Student’s *t*-test was used for comparisons in real-time qPCR, cytotoxicity, and β-galactosidase assays. A *p*-value < 0.05 was considered statistically significant.

## 3. Results

### 3.1. Crc Regulates the Transcription of exsA

**Figure 1:**
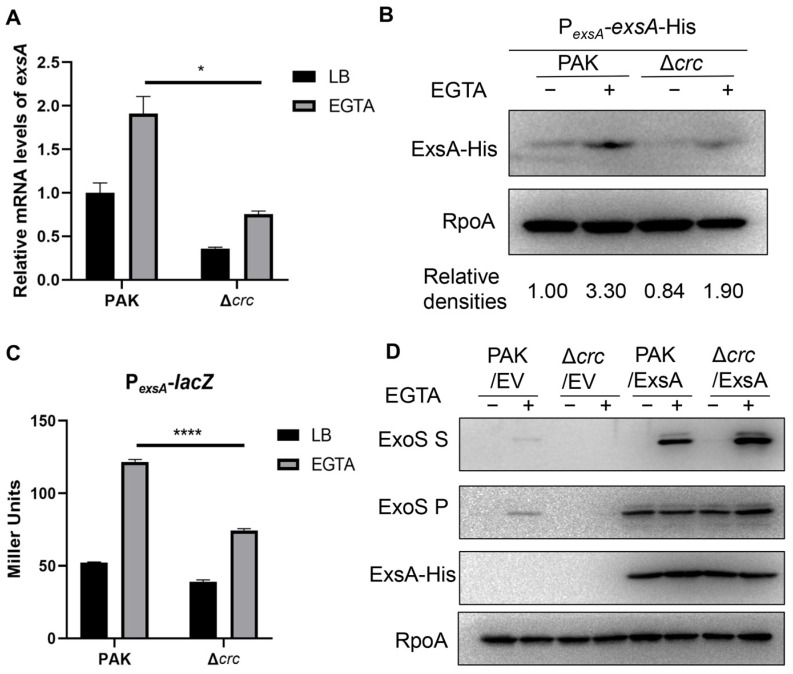
Crc regulates the transcription of *exsA*. (**A**) *exsA* mRNA levels in PAK and the Δ*crc* with or without EGTA were determined by RT-qPCR. The fold changes were calculated relative to the expression level in wild type PAK grown in LB. Data represent the mean ± standard deviation of the results from three samples. *, *p* < 0.05 by Student’s *t*-test. (**B**) Western blot analysis of the ExsA-His levels in samples prepared from PAK/P*_exsA_*-*exsA*-His and Δ*crc*/P*_exsA_*-*exsA*-His strains with or without EGTA. RNA Polymerase α subunit (RpoA) served as a loading control. The density of each band was determined with Image J 1.51J8. Relative densities were determined by using RpoA as the internal control. The data shown represent the results from three independent experiments. (**C**) Promoter activities of *exsA* were determined by a β-galactosidase activity assay after the bacteria containing P*_exsA_*-*lacZ* transcriptional fusion were grown with or without EGTA. Data represent the mean ± standard deviation of the results from three samples. ****, *p* < 0.0001 by Student’s *t*-test. (**D**) Western blot analysis of ExsA-His and ExoS in the supernatants and bacterial pellets from indicated strains grown with or without EGTA. RpoA served as a loading control. The data shown represent the results from three independent experiments. S, supernatant; P, pellet; EV, empty vector.

By using a Δ*crc* mutant in wild type PAK, we verified that Crc is required for the bacterial cytotoxicity and expression of the T3SS effector protein ExoS (Figure S1). Since ExsA functions as a master regulator of the T3SS, we investigated the expression of ExsA. The *exsA* mRNA level was reduced by the mutation of *crc* (Figure 1A). Given that the transcription of *exsA* is driven by both its own promoter and the ExsA-activated *exsC* promoter, we firstly assessed the ExsA expression by using a 6 × His-tagged *exsA* driven by the *exsA* promoter (P*_exsA_*-*exsA*-His). Mutation of *crc* decreased the ExsA levels in the Δ*crc* mutant under T3SS-inducing conditions (Figure 1B). In addition, a β-galactosidase assay with a P*_exsA_*-*lacZ* transcriptional fusion demonstrated reduced *exsA* promoter activity in the Δ*crc* mutant under T3SS-inducing conditions (Figure 1C). Meanwhile, overexpression of ExsA increased the expression of ExoS (Figure 1D). Collectively, these results demonstrate that Crc controls the T3SS by regulating the *exsA* promoter activity.

### 3.2. Crc Regulates ExsA Through the cAMP-Vfr Pathway

**Figure 2:**
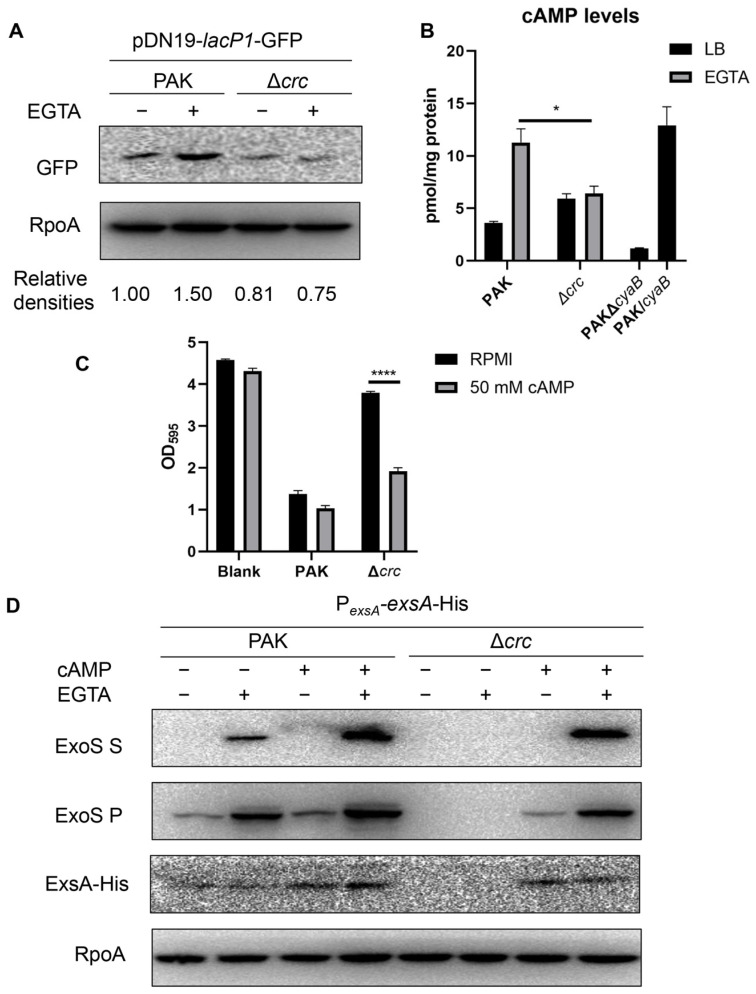
Crc regulates ExsA through the cAMP-Vfr pathway. (**A**) Western blot analysis of GFP driven by *lacP1* promoter from equivalent bacterial cells. RpoA served as a loading control. The density of each band was determined with Image J. Relative densities were determined by using RpoA as the internal control. The data shown represent the results from three independent experiments. (**B**) Intracellular cAMP levels of indicated strains. The cAMP levels of indicated strains were measured by an ELISA Kit. Data represent the mean ± standard deviation of the results from two samples. *, *p* < 0.05 by Student’s *t*-test. (**C**) Bacterial cytotoxicity. A549 cells were infected with wild type PAK and the Δ*crc* mutant at a multiplicity of infection (MOI) of 50 with or without exogenous supplement of 50 mM cAMP. The live cells levels were quantified by crystal violet staining. Data represent the mean ± standard deviation of the results from three samples. ****, *p* < 0.0001 by Student’s *t*-test. (**D**) Expression of ExoS and ExsA-His in indicated strains with or without 5 mM EGTA and 50 mM cAMP. The data shown represent the results from three independent experiments. S, supernatant; P, pellet.

The *exsA* promoter is regulated by multiple transcription factors, including PsrA, MvaT, Fis, and Vfr/cAMP [12,17,18,19]. By using 6 × His-tagged fusions driven by each of their own promoters, we found that the expression of these regulators was not affected by the mutation of *crc* (Figure S2). We then examined the cAMP levels with a *gfp* gene driven by the *lacP1* promoter, which is regulated by the intracellular cAMP level [37]. The GFP level in the Δ*crc* mutant was lower than that in the wild type PAK under T3SS-inducing conditions (Figure 2A). We then determined the intracellular cAMP levels. In *P. aeruginosa*, cAMP is synthesized by CyaA, CyaB, and degraded by CpdA [14,15,16,40]. The cAMP synthesis enzyme CyaB was used as a control. ELISA results demonstrated that the cAMP level was reduced in the Δ*cyaB* mutant and increased in the *cyaB*-overexpressing strain (PAK/*cyaB*) (Figure 2B). In the Δ*crc* mutant, the intracellular cAMP level was reduced under the T3SS-inducing condition (Figure 2B). In addition, supplementation of cAMP increased the expression levels of ExsA and ExoS as well as the cytotoxicity of the Δ*crc* mutant (Figure 2C,D). Collectively, these results suggest that Crc regulates the expression of *exsA* by influencing the intracellular cAMP level.

### 3.3. Crc Regulates the cyaB mRNA Stability

**Figure 3:**
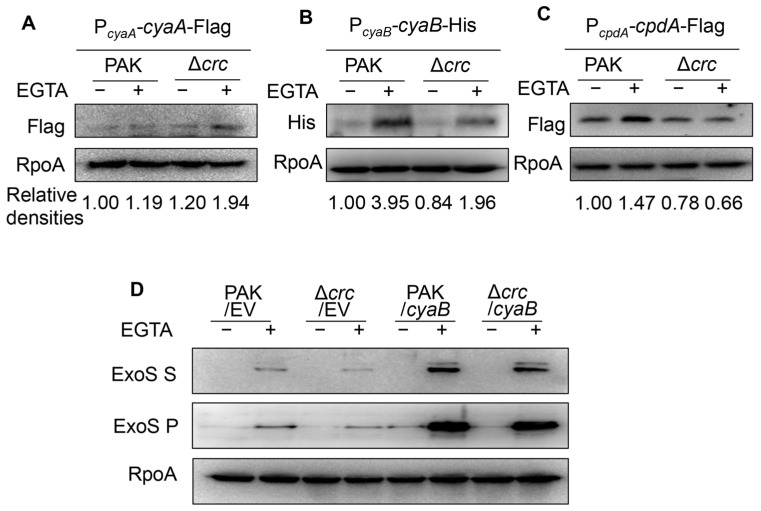
Crc regulates CyaB. (**A**–**C**) Western blot analysis of CyaA (**A**), CpdA (**B**), and CyaB (**C**) in indicated strains grown with or without EGTA. RpoA served as a loading control. The density of each band was determined with Image J. Relative densities were determined by using RpoA as the internal control. The data shown represent the results from three independent experiments. (**D**) Western blot analysis of ExoS in indicated strains grown with or without EGTA. RpoA served as a loading control. The data shown represent the results from three independent experiments. S, supernatant; P, pellet; EV, empty vector.

To understand the mechanism of the reduced cAMP level, we constructed Flag-tagged CyaA, CpdA, and 6 × His-tagged CyaB driven by their native promoters. Under T3SS-inducing conditions, the expression level of CyaA was similar in wild type PAK and the Δ*crc* mutant (Figure 3A), while the expression level of CyaB was lower in the Δ*crc* mutant (Figure 3B). Meanwhile, the CpdA level was reduced in the Δ*crc* mutant under T3SS-inducing conditions, presumably due to the lower cAMP level (Figure 3C), as the expression of *cpdA* is positively regulated by cAMP [16]. We then overexpressed *cyaB* in the bacteria, which resulted in similar levels of ExsA (expressed from the P*_exsA_*-*exsA*-His) and ExoS in the Δ*crc* mutant and wild type PAK (Figure 3D).

**Figure 4:**
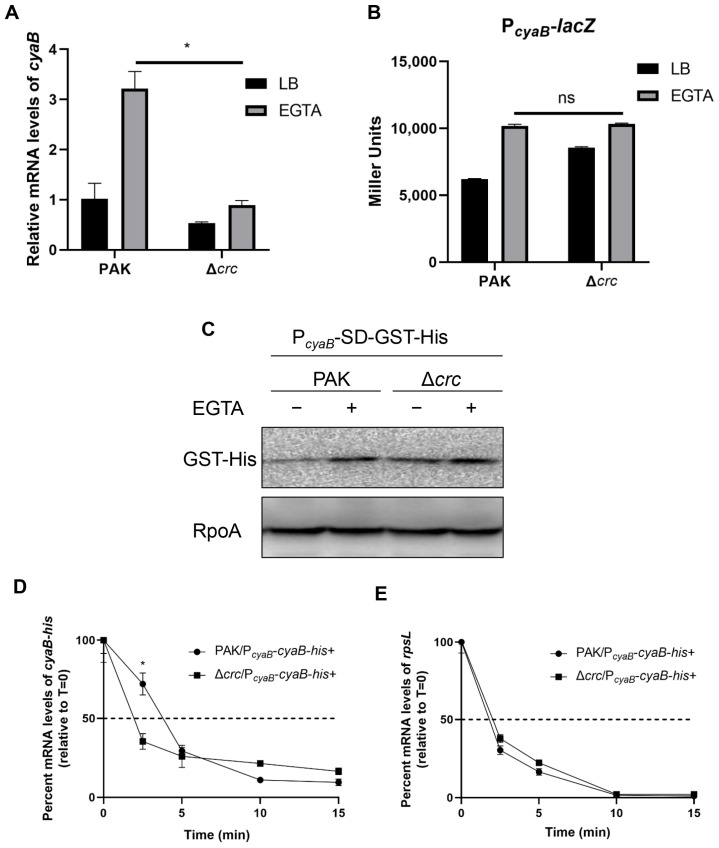
Crc influences the expression of CyaB at the posttranscriptional level. (**A**) Relative quantification of *cyaB* expression in PAK and the Δ*crc* mutant grown with or without EGTA determined by RT-qPCR. The fold changes were calculated relative to the expression level in wild type PAK grown in LB. Data represent the mean ± standard deviation of the results from three samples. *, *p* < 0.05 by Student’s *t*-test. (**B**) Promoter activities of *cyaB* determined by the β-galactosidase activity assay. Wild type PAK and the Δ*crc* mutant carrying the P*_cyaB_*-*lacZ* transcriptional fusion were grown with or without EGTA, followed by the β-galactosidase activity assay. Data represent the mean ± standard deviation of the results from three samples. ns, not significant. (**C**) Western blot analysis of the GST-His in samples prepared from PAK and the Δ*crc* mutant carrying P*_cyaB_*-SD-GST-His grown with or without EGTA. RpoA served as a loading control. The data shown represent the results from three independent experiments. (**D**,**E**) Relative quantification of the *cyaB*-His or *rpsL* mRNAs by RT-qPCR in rifampicin-treated strains under EGTA-inducing conditions. Data represent the percentage of mRNA transcripts relative to the time point when rifampicin was added. The *gfp* mRNA level in each sample was used as the internal control for normalization. Data represent the mean ± standard deviation of the results from three samples. *, *p* < 0.05 by Student’s *t*-test.

To understand the mechanism of CyaB downregulation, we examined the relative mRNA levels of *cyaB* by RT-qPCR. The mRNA level of *cyaB* was lower in the Δ*crc* mutant under T3SS-inducing conditions (Figure 4A). We thus examined the *cyaB* promoter activity by using a *lacZ* transcriptional fusion (P*_cyaB_*-*lacZ*) and a 6 × His tagged GST driven by P*_cyaB_* (P*_cyaB_*-*gst*-His). The expression levels of both LacZ and GST-His were similar in the wild type PAK and the Δ*crc* mutant (Figure 4B,C), demonstrating similar *cyaB* promoter activities. These results indicated a posttranscriptional regulation of *cyaB* by Crc. Thus, we examined the *cyaB* mRNA stability by RT-qPCR. Under T3SS-inducing conditions, mutation of *crc* resulted in a faster degradation of the *cyaB* mRNA (Figure 4D,E), suggesting that Crc affects *cyaB* mRNA stability.

### 3.4. Crc Affects cyaB mRNA Stability Through Its 5′-UTR

Since 5′-UTR has been shown to be involved in regulating its mRNA stability, we investigated the role of the *cyaB* 5′-UTR in the Crc-mediated regulation. A 5′ rapid amplification of cDNA ends (5′ RACE) assay located the transcription start site (TSS) of *cyaB* at 85 bp upstream of its start codon (Figure S3). We replaced the 85 bp 5′-UTR sequence in the P*_cyaB_*-*cyaB*-His with a ribosome binding site from the vector pET28a, resulting in P*_cyaB_*-SD-*cyaB*-His. However, the CyaB-His level was diminished, making it difficult to directly compare the protein levels by western blotting. Thus we enriched the CyaB-His protein by Ni-chromatography. In contrast to the lower CyaB-His level from the P*_cyaB_*-*cyaB*-His in the Δ*crc* mutant, replacing the native 5′-UTR sequence with the exogenous sequence (P*_cyaB_*-SD-*cyaB*-His) resulted in similar levels of CyaB-His in wild type PAK and the Δ*crc* mutant under T3SS-inducing conditions (Figure S4A), indicating a role of the native 85 bp 5′-UTR in regulating the *cyaB* mRNA stability.

**Figure 5:**
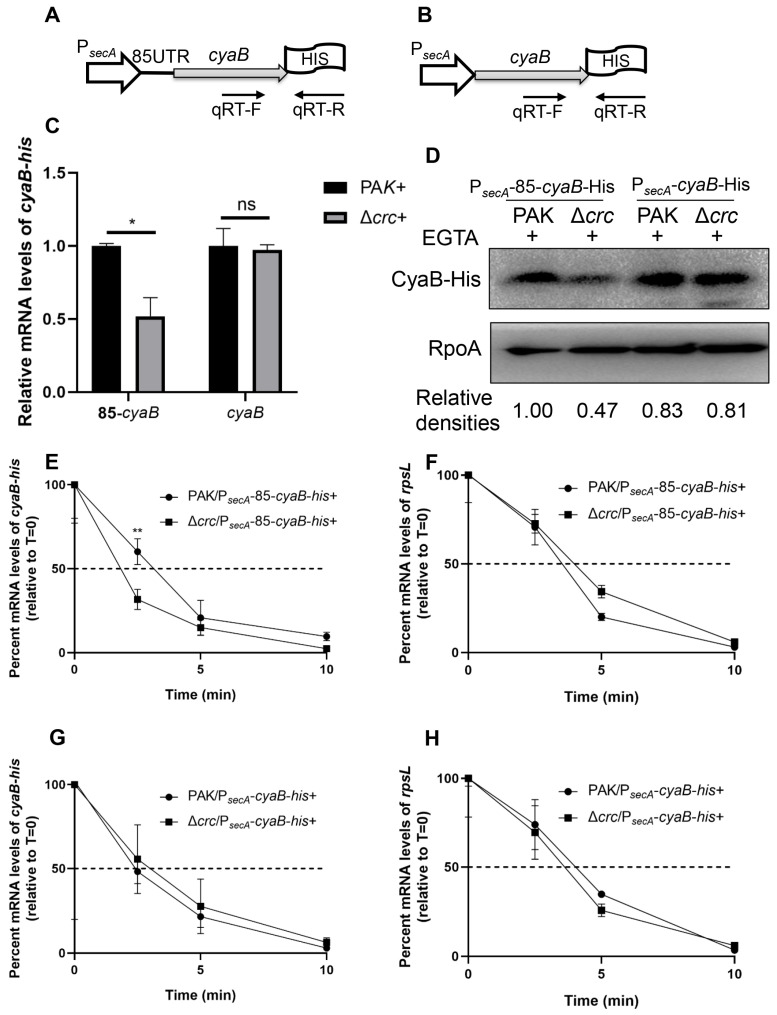
Crc regulates the *cyaB* mRNA stability through its 5′-UTR. Schematic diagram of a *cyaB*-His gene driven by the *secA* promoter with the *cyaB* (**A**) or *secA* (**B**) 5′-UTR. The positions of qPCR primers are indicated by arrows. (**C**) Relative mRNA levels of the *cyaB*-His in PAK and the Δ*crc* mutant grown in the presence of EGTA were determined by RT-qPCR. The fold changes were calculated relative to the expression level in wild type PAK. Data represent the mean ± standard deviation of the results from three samples. *, *p* < 0.05 by Student’s *t*-test; ns, not significant. (**D**) Amounts of CyaB-His. The CyaB-His protein from indicated strains grown with 5 mM EGTA was detected by western blot assay. RpoA served as a loading control. The density of each band was determined with Image J. Relative densities were determined by using RpoA as the internal control. The data shown represent the results from three independent experiments. (**E**–**H**) Relative quantification of the *cyaB-his* or *rpsL* mRNA by RT-qPCR in rifampicin-treated strains under the EGTA inducing condition. Data represent the percentage of mRNA transcripts relative to the time point when rifampicin was added. The *gfp* mRNA level in each sample was used as the internal control for normalization. Data represent the mean ± standard deviation of the results from three samples. **, *p* < 0.01 by Student’s *t*-test.

To verify the result, we utilized a stronger promoter to drive the expression of the *cyaB*-His fusions. A previous study demonstrated that the expression of *secA* is not affected by *crc* mutation [41]. By using RT-qPCR and a C-terminal 6 × His tagged *secA* driven by its native promoter (P*_secA_*-*secA*-His), we demonstrated that the expression of *secA* is similar between wild type PAK and the Δ*crc* mutant under T3SS-inducing conditions (Figure S4B–D). Then we fused the *secA* promoter with the *cyaB*-His with the native and exogenous 5′-UTRs, resulting in P*_secA_*-85-*cyaB*-His and P*_secA_*-*cyaB*-His, respectively (Figure 5A,B). The expression of CyaB-His from P*_secA_*-85-*cyaB*-His was lower in the Δ*crc* mutant under T3SS-inducing conditions, whereas the expression of CyaB-His from P*_secA_*-*cyaB*-His was similar in wild type PAK and the Δ*crc* mutant (Figure 5C,D). In addition, the presence of the native *cyaB* 5′-UTR sequence resulted in lower stability of the *cyaB* mRNA in the Δ*crc* mutant, whereas replacement with the exogenous 5′-UTR sequence resulted in similar mRNA stability in the two strains (Figure 5E–H). In combination, these results demonstrate that Crc regulates *cyaB* mRNA stability through the 5′-UTR region.

## 4. Discussion

**Figure 6:**
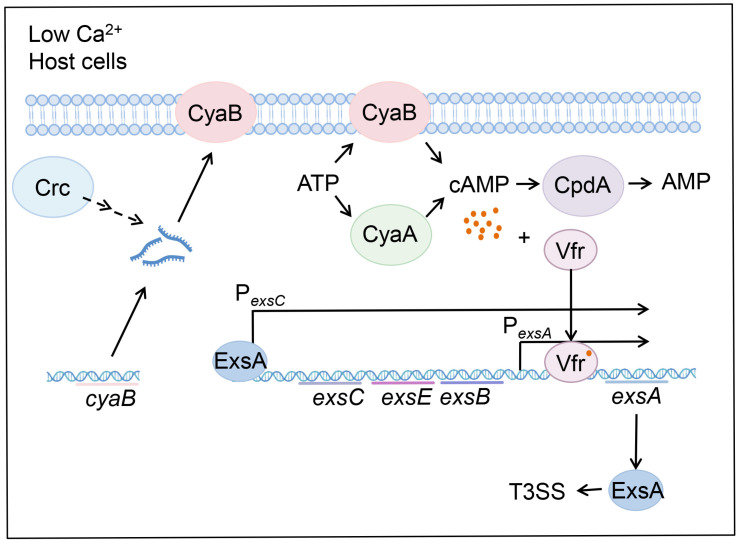
Schematic diagram of the Crc-mediated regulation on the T3SS. In *P. aeruginosa*, CyaA and CyaB synthesize cAMP using ATP as the substrate, CpdA degrades cAMP to AMP. The transcription factor Vfr binds to cAMP and activates the promoter of *exsA*. *exsA* is located in the *exsCEBA* operon, and its transcription is driven by its own adjacent promoter and the *exsC* promoter. ExsA is the central regulator that controls the expression of all the T3SS genes. Under T3SS-inducing conditions, such as contact with host cells and EGTA-caused calcium depletion, Crc affects the expression of CyaB by influencing the mRNA stability, which subsequently affects cAMP levels, thereby impacting the expression of *exsA* and the T3SS genes.

In this work, we demonstrated that mutation of *crc* reduces the *cyaB* mRNA stability under T3SS-inducing conditions, leading to reduced intracellular cAMP level and defective T3SS. We further demonstrated that the 5′-UTR of *cyaB* mRNA is involved in Crc-mediated regulation on the mRNA stability. Based on our results, we propose a regulatory model (Figure 6).

Crc is a global regulator controlling CCR that prevents the utilization of less-preferred carbon sources [42]. In *Escherichia coli*, CCR depends on the phosphotransferase system (PTS) and cAMP-CRP complex [43]. The cAMP levels correspond to glucose availability. In the absence of glucose, the intracellular cAMP concentration is increased, leading to the formation of the cAMP-CRP complex, which activates the alternative catabolic pathways by activating transcription of corresponding genes [44]. Unlike glucose, the preferred carbon source of *E. coli*, certain organic acids and amino acids are preferred carbon sources of *P. aeruginosa* [45]. The hierarchy of carbon source preference in *P. aeruginosa* has been demonstrated. Succinate and malate are at the top of the preferred carbon sources, followed by glucose, citrate or histidine, and then mannitol, oxaloacetate or pyruvate [46,47,48]. Although the *P. aeruginosa* Vfr is 91% similar to the *E. coli* CRP and also binds cAMP, it is not required for CCR [49]. The intracellular cAMP level remains similar in response to different carbon sources, indicating that cAMP is not involved in CCR in *P. aeruginosa* [50]. The distinct CCR mechanisms in *E. coli* and *P. aeruginosa* might reflect their adaptation strategies to different ecological niches. For *E. coli*, the CRP-cAMP mechanism aligns with the feast–famine cycles of the gut, switching to alternative catabolic pathways upon glucose depletion to optimize resource capture [51,52,53]. Conversely, for *P. aeruginosa* in its nutrient-poor environmental niche, the Crc/Hfq system ensures metabolic efficiency by directly repressing the synthesis of enzymes for less favorable substrates when preferred carbon sources are present [54,55]. Meanwhile, the utilization of organic acids is not only an adaptation to nutrient availability but also a metabolically efficient strategy. These organic acids can be directly utilized in the TCA cycle for energy production or as biosynthetic precursors [45,54,56].

Crc usually functions together with the RNA chaperone Hfq, regulating gene expressions at the posttranscriptional level [20]. Initially, Crc is considered to directly bind to CA rich regions of mRNAs [57,58]. However, further studies have shown that Crc does not directly bind RNA. Instead, Hfq is the protein that binds target mRNAs [23,59]. For example, the aliphatic amidase gene *amiE* is a typical CCR-regulated gene, whose translation is inhibited by Hfq and Crc [23,24,58,60,61]. The Lon protease mRNA was bound by the complex which represses its translation [28]. Mutation of *crc* increases the amount of Lon protease, leading to excessive degradation of RhlI and subsequent attenuation of the quorum sensing system [28]. The sRNA CrcZ relieves CCR by binding to Hfq [58]. CrcZ is directly regulated by the two-component system CbrA/B in response to extracellular carbon sources [25]. Besides CrcZ, a CrcA protein interacts with Crc and prevents the formation of the Hfq-Crc complex on target mRNA [62].

Combined transcriptomic and proteomic analyses revealed around 200 Hfq-Crc targets [41,63]. Besides catabolite repression, Crc is involved in the regulation of iron uptake, c-di-GMP metabolism, oxidative stress response, etc. [41,60]. By using a ChIPPAR-seq assay, Kambara et al. identified the potential direct targets of Hfq/Crc, a set ranging from carbon metabolic functions to virulence regulators, which further supports the pleiotropic effects of Crc in coordinating the carbon source utilization and virulence factor expression [64].

Based on the function of Crc, we explored the regulatory mechanism of Crc on the T3SS. We found that in the absence of EGTA, mutation of *crc* slightly increased the cAMP level. However, the presence of EGTA increased the cAMP level in the wild type strain but not in the Δ*crc* mutant (Figure 2B). We speculated that, as a catabolite repression control protein, mutation of *crc* may affect bacterial metabolism, leading to increased cAMP in the LB medium. Meanwhile, Crc is involved in bacterial response to the T3SS inducing signal, such as EGTA-caused calcium depletion. Further research is needed to explore the role of Crc in gene regulation under T3SS-inducing conditions.

CyaB is a member of the class III adenylyl cyclase that produces the secondary messenger molecule cAMP [14,65]. In *P. aeruginosa*, CyaB rather than CyaA produces the majority of cAMP [14,66]. At the transcriptional level, *cyaB* is upregulated under T3SS-inducing conditions (EGTA treatment) [14]. However, the signaling mechanism and regulatory genes remain to be elucidated. At the posttranslational level, FimL modulates the CyaB activity [37,67]. It has been demonstrated that c-di-GMP negatively regulates cAMP level [68]. Further studies have demonstrated that c-di-GMP does not affect the expression of CyaA, CyaB or their activities [68]. Here in this work, we demonstrated that the *cyaB* mRNA stability is regulated by Crc under T3SS-inducing conditions. We further demonstrated that the 5′-UTR region of *cyaB* is involved in the regulation. Collectively, the expression of CyaB is regulated at multiple levels, which indicates a delicate regulation on the critical messenger molecule cAMP. It is worth noting that the 5′-UTR was also included in the experiments when we detected the promoter activity by using *lacZ* and *gst* transcriptional fusions, but no difference was observed between wild type PAK and the Δ*crc* mutant (Figure 4B,C). We suspect that the presence of exogenous ribosome binding site sequences in front of the reporter genes might affect the secondary structure of the *cyaB* 5′-UTR, thereby altering its stability. The mRNA stability is regulated by multiple mechanisms. Various nucleases are involved in mRNA degradation, such as endoribonucleases (RNase E and RNase III), exoribonucleases (PNPase and RNase R), and oligoribonucleases (Orn) [69]. The mRNA sequence and/or structure affects its stability by influencing the accessibility of nucleases [70]. sRNAs are involved in regulating mRNA stabilities in response to environmental cues. For instance, the sRNA PhrS stimulates synthesis of the quinolone signal through activating the translation of PqsR in response to oxygen availability [71]. Another sRNA PqsS binds to the *pqsL* mRNA and destabilizes it through recruiting RNase E [72]. Considering that Crc is a posttranscriptional regulator, it is likely that Crc regulates the expression of CyaB through another protein or sRNA. Further research is needed to investigate the mechanism of Crc-mediated regulation on the *cyaB* mRNA stability.

On the other hand, while observing a decrease in CyaB and cAMP, we also observed a decrease in CpdA, which confirms that the expression of *cpdA* is positively regulated by cAMP in couple with Vfr [16]. This feedback mechanism contributes to the homeostasis of cAMP in *P. aeruginosa* [16]. Our results demonstrated reduction of the CyaB protein level under T3SS-inducing conditions, which might be the major cause that results in the defective T3SS gene expression. However, it is possible that Crc regulates the expression of *cpdA* through a cAMP independent pathway. Further studies are warranted to examine the possibility.

Disturbances in carbon metabolism affect bacterial virulence through various pathways. For example, mutation of the isocitrate lyase gene *aceA* results in defective T3SS gene expression [73]. The glucose transport regulator GltR is involved in the regulation of the T3SS genes [74]. How *P. aeruginosa* balances metabolism and virulence remains unclear. One possible reason is that metabolic changes alter bacterial energy levels. In response, bacteria might coordinate the expression of the energy-costly virulence determinants and survival. The regulation of Crc on the T3SS further verify the interrelationship between bacterial carbon metabolism and virulence. These results indicate that the carbon metabolism pathway can be targeted to inhibit bacterial virulence gene expression. Inhibitors targeting Crc-mediated regulatory pathways or metabolism genes could decrease bacterial virulence without exerting the selection pressure as antibiotics. Such strategy presents a promising approach to delay the development of resistance. In addition, these inhibitors may achieve synergistic effect with antibiotics in the treatment of bacterial infections.

## 5. Conclusions

In this work, we demonstrated the role of Crc in regulating the *cyaB* mRNA stability under inducing conditions, which affects cAMP production and subsequent expression of T3SS genes. Our results shed light on the complex regulatory network of the T3SS in response to environmental signals. Further research is needed to elucidate the mechanisms by which Crc regulates mRNA stability.

## Data Availability

The original contributions presented in this study are included in the article/Supplementary Material. Further inquiries can be directed to the corresponding author.

## Supplementary Materials

The following supporting information can be downloaded at: https://www.mdpi.com/article/10.3390/microorganisms13112587/s1, Figure S1: Crc influences the T3SS; Figure S2: Expression levels of the transcription factors; Figure S3: Identification of the transcription start site of cyaB; Figure S4: The CyaB expression level; Table S1: Bacterial strains and plasmids used in this study; Table S2: Primers used in this study.

## Abbreviations

The following abbreviations are used in this manuscript:

EV	Empty vector
UTR	Untranslated Regions
T3SS	Type III Secretion System
