# Supplementary material for: The Catabolite Repression Control Protein Crc Regulates the Type III Secretion System Through the Adenylate Cyclase CyaB in Pseudomonas aeruginosa
Source: Microorganisms. 2025 Nov 13;13(11):2587. doi: 10.3390/microorganisms13112587 (PMC12654477; doi:10.3390/microorganisms13112587)
Supplement: Supplementary file 1 [file microorganisms-13-02587-s001.zip › microorganisms-3890977-supplementary.pdf]

Figure S1

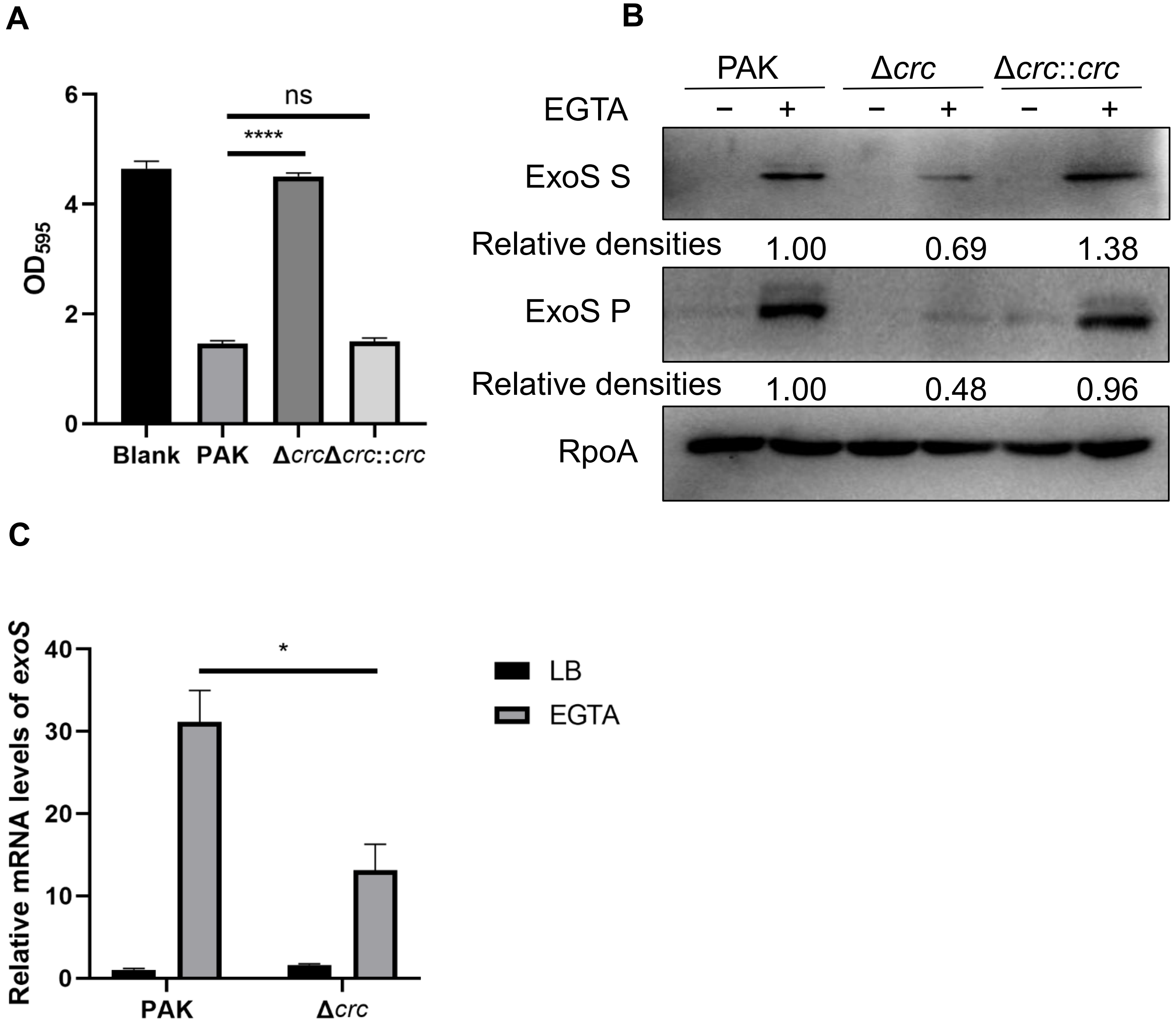

Figure S1. Crc influences the T3SS. (A) Cytotoxicity of PAK, Δ*crc* and the complemented strain. A549 cells were infected with the indicated strains at a multiplicity of infection (MOI) of 50. The live cells were stained with crystal violet after infection. The cell associated crystal violet was dissolved and quantified by measuring OD_{595}. Data represent the mean ± standard deviation of the results from three samples.****, $P<0.0001$ by Student's *t*-test; ns, not significant. (B) Western blot analysis of ExoS in bacteria grown with or without EGTA. The RNA Polymerase α subunit (RpoA) served as a loading control. The density of each band was determined with Image J. Relative densities were determined by using RpoA as the internal control. The data shown represent the results from three independent experiments. (C) Relative amounts of *exoS* mRNA in PAK and the Δ*crc* mutant grown with or without EGTA were determined by RT-qPCR. Data represent the mean ± standard deviation of the results from three samples. *, $P<0.05$ by Student's *t*-test.

Figure S2

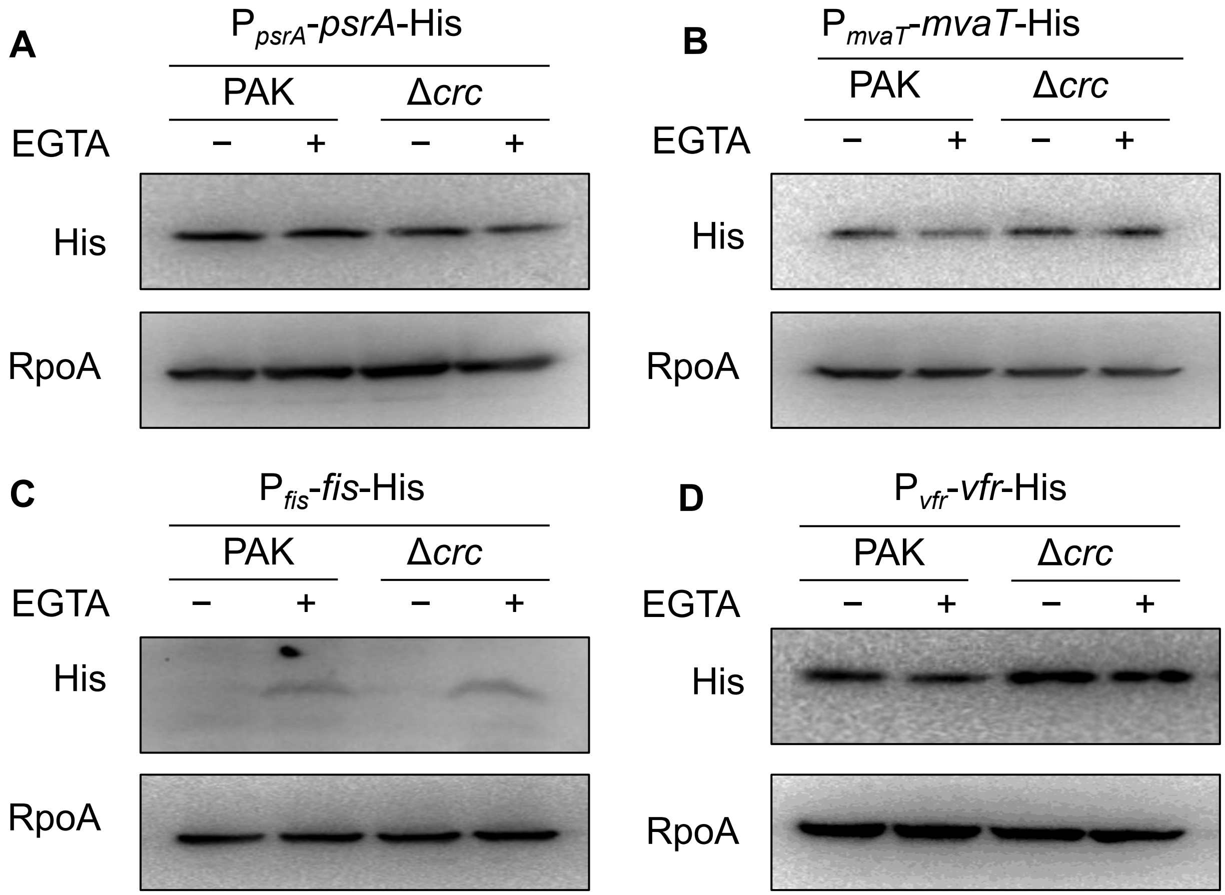

Figure S2. Expression levels of the transcription factors. Western blot analysis of PsrA (A), MvaT (B), Fis (C) and Vfr (D) in samples preparation indicated strains with or without EGTA. RpoA served as a loading control. The data shown represent the results from three independent experiments.

Figure S3

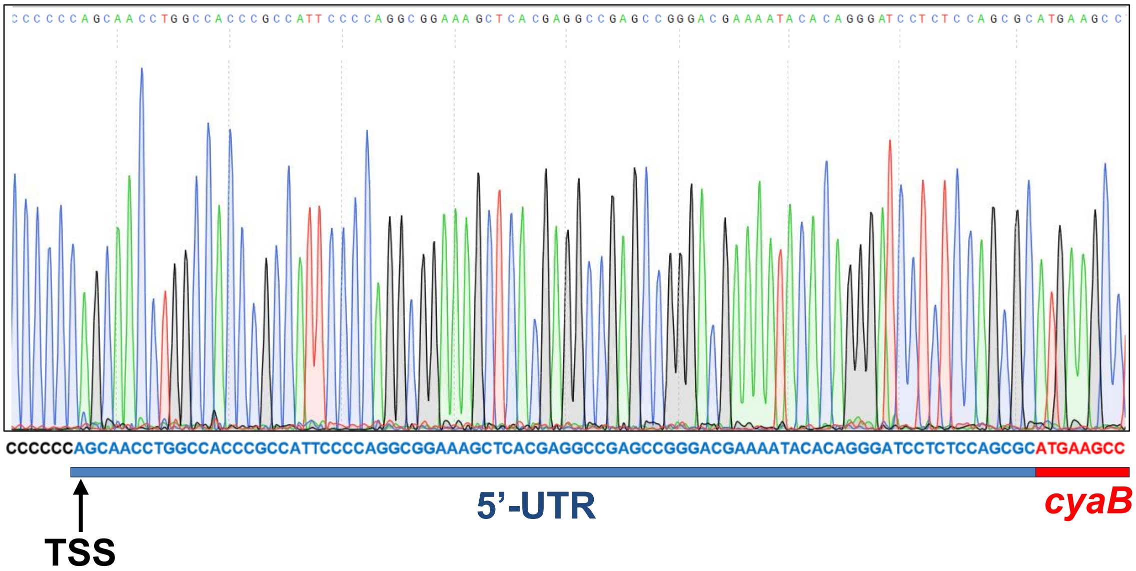

Figure S3. Identification of the transcription start site of *cyaB*. The transcription start site of *cyaB* was identified by 5'-RACE. The upper panel shows the sequencing result of the *cyaB* 5'-RACE product. In the lower panel, the 5'-UTR region was shown in blue and *cyaB* coding region was shown in red. The position of the transcription start site (TSS) is indicated by a black arrow.

Figure S4

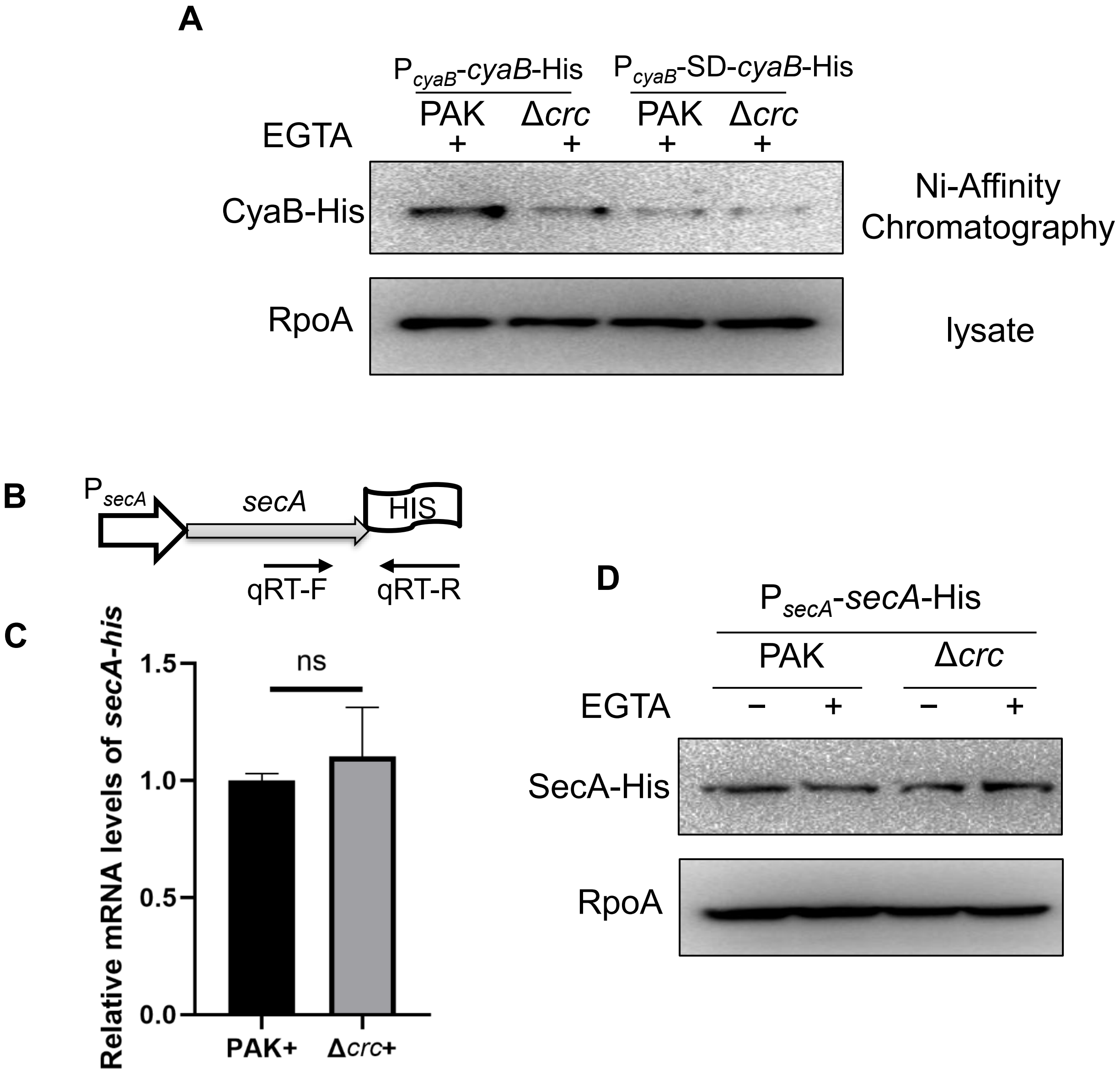

Figure S4. The CyaB expression level. (A) Western blot analysis of purified CyaB-His proteins preparation from indicated strains in the presence of EGTA. RNA Polymerase α subunit (RpoA) from bacterial lysate served as a loading control. The data shown represent the results from three independent experiments. (B) Schematic diagram of a *secA*-His gene driven by the secA promoter. The positions of qPCR primers are indicated by arrows. (C) Relative *secA*-His mRNA levels in bacteria grown with EGTA were determined by RT-qPCR. ns, not significant. (D) The SecA-His and RpoA levels were determined by western blot assay.

Table S1. Bacterial strains and plasmids used in this study.

Strains or plasmids	Description	Source or reference
**strains**		
DH5α	F^- ϕ 80d*lacZ*ΔM15 *endA1 recA1 hsdR17*(r_K^- m_K^+) *supE44 thi-1 relA1* Δ(*lacZYA-argF*)*U169 gyrA96 deoR*	TransGen
S17-1	RP4-2 Tc::Mu Km::Tn*7* Tp^r Sm^r Pro Res^- Mod^+	Stratagene
PAK	Wild type *P. aeruginosa* strain	David Bradley
PAKΔ*crc*	PAK with *crc* gene deleted	This study
PAKΔ*crc*::*crc*	PAKΔ*crc* with *crc* inserted on chromosome	This study
**Plasmids**		
pEX18Tc-*crc*	*crc* gene deletion on pEX18Tc; Tc^r	This study
pUC18T-mini-Tn7T-*crc*	*crc* gene from PAK in pUC18T-mini-Tn7T; Gm^r	This study
pUCP20-P_{exsA}-*exsA*-Flag-His	Flag-His-tagged *exsA* gene from PAK in promoterless pUCP20; Amp^r	[1]
pUCP20-P_{cyaA}-*cyaA*-Flag	Flag-tagged *cyaA* gene from PAK in promoterless pUCP20; Amp^r	[2]
pUCP20-P_{cyaB}-*cyaB*-Flag-His	Flag-His-tagged *cyaB* gene from PAK in promoterless pUCP20; Amp^r	This study
pUCP20-P_{cyaB}-SD-*cyaB*-His	His-tagged *cyaB* gene fused with rbs from pET28a driven by *cyaB* promoter in promoterless pUCP20; Amp^r	This study
pUCP20-P_{cpdA}-*cpdA*-Flag	Flag-tagged *cpdA* gene from PAK in promoterless pUCP20; Amp^r	[2]
pUCP20-*cyaB*	*cyaB* gene from PAK in pUCP20; Amp^r	This study
pUCP20-P_{cyaB}-SD-GST- His	His-tagged *gst* gene fused with rbs from pET28a driven by *cyaB* promoter in promoterless pUCP20; Amp^r	This study
pUCP20-P_{psrA}-*psrA*-His	His-tagged *psrA* gene in promoterless pUCP20; Amp^r	This study
pUCP20-P_{mvaT}-*mvaT*-His	His-tagged *mvaT* gene in promoterless pUCP20; Amp^r	This study
pUCP20-P_{fis}-*fis*- His	His-tagged *fis* gene in promoterless pUCP20; Amp^r	This study
pUCP20-P_{vfr}-*vfr*- His	His-tagged *vfr* gene in promoterless pUCP20; Amp^r	This study
pDN19-P_{exsA}-*lacZ*	*exsA* promoter fused to promoterless *lac*Z on pDN19*lac*ZΩ; Sp^r, Sm^r, Tc^r	[1]
pDN19-P_{cyaB}-*lacZ*	*cyaB* promoter fused to promoterless *lac*Z	[1]

	on pDN19*lac*ZΩ; Sp^r, Sm^r, Tc^r	
pDN19-*lacP1*-GFP	*lacP1* promoter of *E. coli* fused to promoterless *gfp* on pDN19*lac*ZΩ; Sp^r, Sm^r, Tc^r	This study
pMMB76EH-*exsA*-His	His-tagged *exsA* gene from PAK in pMMB67EH; Amp^r	This study
pUCP20-P_{secA}-*secA*-His	His-tagged *secA* gene from PAK in promoterless pUCP20; Amp^r	This study
pUCP20-P_{secA}-*cyaB*-His	His-tagged *cyaB* gene fused with P_{secA} in promoterless pUCP20; Amp^r	This study
pUCP20-P_{secA}-85-*cyaB*-His	His-tagged *cyaB* gene with 5'UTR fused with P_{secA} in promoterless pUCP20; Amp^r	This study

1. Deng, X., et al., *Fis Regulates Type III Secretion System by Influencing the Transcription of exsA in Pseudomonas aeruginosa Strain PA14.* Front Microbiol, 2017. **8**: p. 669.
2. Jin, Y., et al., *NrtR Regulates the Type III Secretion System Through cAMP/Vfr Pathway in Pseudomonas aeruginosa.* Front Microbiol, 2019. **10**: p. 85.

Table S2. Primers used in this study.

Primer[a]	Sequence 5'-3'[b]	Use
crc-UF	CGGAATTCGCGGTACGGTGCGGATAACC	pEX18Tc-*crc*
crc-UR	GCTCTAGACATGCGGCTTGGTGTCATTC	
crc-DF	GCTCTAGACTTTTCTATTTCCCGTGCGG	
crc-DR	CCAAGCTTTTCGGCGATACCAAGGTGAT	
mini-*crc*-F	GGGGTACCGATGATCTGCATCACTTCGCGGATC	pUC18T-mini-Tn7T-*crc*
mini-*crc*-R	CCAAGCTTTCAGATGCTCAACTGCCAGTCGTAG	
exsA-His-F	CGGAATTCATGCAAGGAGCCAAATC	pMMB67EH-*exsA*-His
exsA-His-R	CCAAGCTTTCAATGGTGATGGTGATGATGGTTATTTTTAGCCCG	
cyaB-Flag-His-F	CCAAGCTTAGTACCCGCTGTGGTCGTTC	pUCP20-P_{cyaB}-*cyaB*-Flag-His
cyaB-Flag-His-R	CGGAATTCTTAATGGTGATGGTGATGATGCTTGTCATCGTCGTCCTTGTAATCGAGGATGACCTTGTCGC	
cyaB-F	CGCGGATCCCGAGCCGGTCCGTCGC	pUCP20-*cyaB*
cyaB-R	CCCAAGCTTTTAGAGGATGACCTTGTCGCGC	
P_{cyaB}-F	CGGAATTCGCCGCCTGCACTACGTCG	pUCP20-P_{cyaB}-SD-GST-His
ol-P_{cyaB}-SD-R	AGGGGACATGGTATATCTCCTTGCGCTGGAGAGGATCCCT	
ol-P_{cyaB}-SD-F	CTCCAGCGCAAGGAGATATACCATGTCCCCTATACTAGGTTA	
GST-His-R	TCACGCCGAGGAAGCTGTTGGCGTCCAGCTGCGAGAT	
ol-PcyaB-85-R	CTGGTATATCTCCTTGCGCTGGAGAGGATCCC	pUCP20-P_{cyaB}-SD-*cyaB*-His
ol-PcyaB-85-cyaB-F	GCAAGGAGATATACCATGAAGCCTACCCTCCCC	
P_{secA}-F	CGAGCTCCTCGCCGTTCGGGCGCCGGG	pUCP20-P_{secA}-*secA*-His
secA-His-R	CGGGATCCTTAATGGTGATGGTGATGATGGTCCAGCTGCCCGTGGC	
ol-P_{secA}-*cyaB*-R	TCGGGGAGGGTAGGCTTCATATCGACTTGGTCATCCACAC	pUCP20-P_{secA}-*cyaB*-His
ol-P_{secA}-*cyaB*- -F	GTGTGGATGACCAAGTCGATATGAAGCCTACCCTCCCCGA	
cyaB-His-R	CGGGATCCTTAATGGTGATGGTGATGATGGAGGATGACCTTGTCGCGCAG	
ol-P_{secA}-85-*cyaB*-R	TGGCGGGTGGCCAGGTTGCTATCGACTTGGTCATCCACAC	pUCP20-P_{secA}-85-*cyaB*-His
ol-P_{secA}-85-*cyaB*-F	GTGTGGATGACCAAGTCGATAGCAACCTGGCCACCCGCCA	
psrA-F	CGGAATTCGCGGCGACCCGTCCG	pUCP20-P_{psrA}-*psrA*-His

psrA-R	CCAAGCTTTCAATGGTGATGGTGATGATGGGCCTTGGCGGGCGTCT	
mvaT-F	CGGGATCCTGATTCGCCATTCCATGGAGAATTCGCG	pUCP20-P_{mvaT}-*mvaT*-His
mvaT-R	CCAAGCTTTTAATGGTGATGGTGATGATGGCCGAGCAGGGTGGCCC	
fis-F	CGGGATCCATCCAGGCTCTTGCGGTGCA	pUCP20-P_{fis}-*fis*-His
fis-R	CCAAGCTTTTAATGGTGATGGTGATGATGAAGAAGATCGTATTGCTTGAG	
vfr-F	CGGAATTCCGGCCTCGAGGAAGGCTTCG	pUCP20-P_{vfr}-*vfr*-His
vfr-R	CCAAGCTTTCAATGGTGATGGTGATGATGGCGGGTGCCGAAGACC	
5-AP	CGACTTGAACTCCACCCCCCCCCCC	for 5'RACE
RACE-*cyaB*-R	ATACCAGCAAGGCCATGCCGA	
5-NP	CGACTTGAACTCCACC	
RACE-*cyaB*-R2	GATATAGGCCAGCACGCGCGAA	
q-*exoS*-F	GCATATTCAATCGCTTCAG	RT-qPCR of *exoS*
q-*exoS*-R	CCTCAATCTGTCCCAAAC	
q-*exsA*-F	GCTATGTCGTAAGTACCA	RT-qPCR of *exsA*
q-*exsA*-R	GAAGCCTTGTAGAAACTG	
q-*exsC*-F	ATGGATTTAACGAGCAAGGTCAA	RT-qPCR of *exsC*
q-*exsC*-R	GAGGGACAGGGAAGGCAAA	
q-*rpsL*-F	CAAAACTGCCCGCAACGT	RT-qPCR of *rpsL*
q-*rpsL*-R	TTTCGGCGTGGTGGTGTAT	
q-*cyaB*-F	GCTCACCGTGTTCTTCTCCG	RT-qPCR of *cyaB*
q-*cyaB*-R	CCGAAGAACACCATGACGCA	
qq-cyaB-F	CAGATCGCGGTCAAGGGCT	RT-qPCR of *cyaB-his*
q-secA-F	CTTCCCGAGGGCCCGGCT	RT-qPCR of *secA-his*
q-*his*-R	TCAGTGGTGGTGGTGGTGGTG	
q-gfp-F	ATGCCTGAAGGTTATGTA	RT-qPCR of *gfp*
q-gfp-R	TGTGAGTTATAGTTGTATTCC	

a: F: forward; R, reverse; U, upstream of specific gene; D, downstream of specific gene; b: The underlines are the sites of restriction enzymes or tag sequences.
